# How education and racial segregation intersect in neighborhoods with persistently low COVID-19 vaccination rates in Philadelphia

**DOI:** 10.1186/s12889-022-13414-3

**Published:** 2022-05-25

**Authors:** John A. Rich, Edward J. Miech, Usama Bilal, Theodore J. Corbin

**Affiliations:** 1 Department of Health Management and Policy, Center for Nonviolence and Social Justice, Dornsife School of Public Health, Drexel University, 1505 Race Street, MS 1047, 6th floor, Philadelphia, PA 19102 USA; 2 Regenstrief Institute, Center for Health Services Research, 1101 West 10th Street, Indianapolis, IN 46202 USA; 3 Department of Epidemiology and Biostatistics, Urban Health Collaborative, Dornsife School of Public Health, Drexel University, 3600 Market St. Suite 730, Philadelphia, PA 19104 USA; 4 Department of Emergency Medicine, Rush University Medical Center, 1750 W. Harrison Street, Suite 108 Kellogg, Chicago, IL 60612 USA

**Keywords:** COVID-19 vaccination, Residential racial segregation, Education, Health inequity, Configurational comparative methods, Coincidence analysis, Geospatial analysis, Intersectionality

## Abstract

**Background:**

COVID-19 infection has disproportionately affected socially disadvantaged neighborhoods. Despite this disproportionate burden of infection, these neighborhoods have also lagged in COVID-19 vaccinations. To date, we have little understanding of the ways that various types of social conditions intersect to explain the complex causes of lower COVID-19 vaccination rates in neighborhoods.

**Methods:**

We used configurational comparative methods (CCMs) to study COVID-19 vaccination rates in Philadelphia by neighborhood (proxied by zip code tabulation areas). Specifically, we identified neighborhoods where COVID-19 vaccination rates (per 10,000) were persistently low from March 2021 – May 2021. We then assessed how different combinations of social conditions (pathways) uniquely distinguished neighborhoods with persistently low vaccination rates from the other neighborhoods in the city. Social conditions included measures of economic inequities, racial segregation, education, overcrowding, service employment, public transit use, health insurance and limited English proficiency.

**Results:**

Two factors consistently distinguished neighborhoods with persistently low COVID-19 vaccination rates from the others: college education and concentrated racial privilege. Two factor values together – low college education AND low/medium concentrated racial privilege – identified persistently low COVID-19 vaccination rates in neighborhoods, with high consistency (0.92) and high coverage (0.86). Different values for education and concentrated racial privilege – medium/high college education OR high concentrated racial privilege – were each sufficient by themselves to explain neighborhoods where COVID-19 vaccination rates were not persistently low, likewise with high consistency (0.93) and high coverage (0.97).

**Conclusions:**

Pairing CCMs with geospatial mapping can help identify complex relationships between social conditions linked to low COVID-19 vaccination rates. Understanding how neighborhood conditions combine to create inequities in communities could inform the design of interventions tailored to address COVID-19 vaccination disparities.

**Supplementary Information:**

The online version contains supplementary material available at 10.1186/s12889-022-13414-3.

## Background

The COVID-19 pandemic has revealed many forms of inequity. For example, it is well-documented that Black and brown communities and poor communities have had higher rates of COVID-19 infection [1, 2]. Geospatial analyses have uncovered racial and socioeconomic inequities in COVID-19 testing (total number of tests/total population), positivity (positive tests/total number of tests), confirmed cases (new cases/total population) and mortality (new deaths/total population) in New York, Philadelphia, and Chicago [3, 4]. These same populations should have been considered as priority populations during the early months of vaccine rollout [5], and this was part of the recommendation of the National Academies of Sciences, Engineering, and Medicine [6]. However, in many cities, communities with higher social vulnerability and higher rates of COVID-19 infections were not prioritized for COVID-19 vaccination [7–9]. For example, COVID-19 vaccination rates in Philadelphia vary widely by neighborhood with COVID-19 vaccination rates being generally lower in socially disadvantaged neighborhoods [10, 11].

Correlational analyses looking at spatial inequities have been able to show that factors such as lack of health insurance, poverty and racial segregation are independently associated with lower rates of COVID-19 vaccination [3, 12–14]. However, since social conditions are highly collinear with each other, approaches using the Social Vulnerability Index (SVI), which incorporates multiple social determinants of health into a single measure, have also been applied to local data [4, 7, 8, 15]. These studies have shown high correlations between the SVI and COVID-19 testing, positivity, confirmed cases and mortality at the neighborhood level [4], and with COVID-19 vaccination rates at the county level [7–9] and the neighborhood level [10]. However, the practical application of studies using multidimensional indices is complex because there are multiple variables involved in the creation of these indices (15 in the case of the SVI); and it is not possible to tease out the impact of individual factors. This fact complicates creating accessible explanations for policymakers and other stakeholders, as interpreting multidimensional indices in terms of policy solutions is challenging. To address these issues, we chose methods that acknowledge the complexity of multiple interacting factors without reducing them to a single dimension.

Ragin and Fiss have proposed addressing the intersectional nature of inequity through using configurational comparative methods (CCMs) which “shift the focus from the separate effects of, for example, race and gender, towards their combined and synergistic effects [16].” CCMs represent a novel approach to understanding complex phenomena. CCMs seek to uncover Boolean conjunctions - the ways in which specific conditions, when they appear together with certain other specific conditions, link directly to important outcomes. CCMs – which include qualitative comparative analysis and coincidence analysis (CNA) – draw on Boolean algebra and set theory to produce configurational models that can complement those generated with more traditional approaches, yielding new and useful insights [17–19].

CCMs are particularly well-suited to understanding geospatial inequities in vaccinations because they are case-based methods which can be applied to a relatively small number of cases, typically between 10 and 100 cases. CCMs can also identify Boolean disjunctions, where multiple pathways lead to the same outcome of interest. These features make CCMs a good fit for analyses focused on exploring and understanding the complex causes of COVID-19 inequities [18, 20, 21].

In this study, we sought to understand how different combinations of social conditions could help explain COVID-19 vaccination levels at the neighborhood level. Our specific goal was to use CCMs to examine configurations of neighborhood social conditions consistently linked to low COVID-19 vaccination rates in Philadelphia. We used CNA, a new cross-case method, to identify the crucial difference-making conditions for the outcome of low COVID-19 vaccination rates [19, 22]. CCMs, including CNA, are increasingly being used in health services research and implementation and dissemination research [23–27].

## Methods

We used data on COVID-19 vaccination rates per 10,000 residents (at least 1 dose) at the zip code tabulation area (ZCTA) level (henceforth, neighborhoods) publicly available from the Philadelphia Department of Public Health (PDPH) on three dates: March 18th, April 18th and May 18th, 2021. For reference, Philadelphia opened eligibility to all adults on April 16th, 2021. We used neighborhood-level data publicly available from the 2014–2018 American Community Survey for sociodemographic variables - % with college education, % uninsured, % households with limited English proficiency, % working in service jobs, % using public transportation, and % overcrowded households [> 1 person per room]. Based on Krieger [28], we also computed the Index of Concentration at the Extremes for Black non-Hispanic populations and for income, also at the neighborhood level.

### Calibration

We performed multi-value calibration on all factors and the outcome COVID-19 vaccination rates. Vaccination rates were calibrated based on PDPH reporting categories for percent of residents with at least one COVID-19 vaccination. For the three dates March 18, April 18 and May 18, we calibrated neighborhood level COVID-19 vaccination rates based on the tertile categories used by the PDPH to publicly report neighborhood rates per 10,000 residents. For March: < 1500 = Low, 1501–2000 = Medium, > 2000 = High; for April: < 2500 = Low, 2501–3400 = Medium, > 3400 = High; and for May: < 3400 = Low, 3401–4500 = Medium, > 4500 = High. Calibration of social conditions (% with college education, % uninsured, % households with limited English proficiency, racial segregation and economic inequity, % working in service jobs, % using public transportation, % overcrowded households) was based on tertiles where values greater than or equal to 67th percentile were categorized as high, values greater than 33rd percentile and less than 67th percentile were categorized as medium, and values less than or equal to 33rd percentile were categorized as low. Because our analysis focused on those neighborhoods with persistently low COVID-19 vaccination rates over the three-month period, our main analysis only includes neighborhoods that had low values in each of the three months, resulting in a total of *n* = 13 neighborhoods out of a total of 43 in the analysis.

### Income inequities: index of concentrations at the extremes-income

To proxy income inequities, we used the Index of Concentration at the Extremes-Income (ICE-Income) measure where negative one (− 1) represents the least concentrated economic privilege (extreme concentration of low-income residents) and positive one (1) represents the most concentrated economic privilege (extreme concentration of high-income residents.) We calibrated ICE-Income based on tertiles where values from − 1 to − 0.23 were coded as zero (low concentrated economic privilege), values from > − 0.23 to < 0.07 were coded as one (medium concentrated economic privilege), and values from 0.07 to 1 were coded as two (high concentrated economic privilege).

### Racial segregation: index of concentrations at the extremes-non-Hispanic Black

To proxy racial segregation, we used the Index of Concentration at the Extremes-Non-Hispanic Black (ICE-BlackNH). ICE-BlackNH values range from negative one (− 1) representing the least concentrated racial privilege (extreme concentration of Black non-Hispanic residents) to positive one (1) representing the most concentrated racial privilege (extreme concentration of White non-Hispanic residents). We calibrated ICE-BlackNH based on tertiles where values from − 1 to − 0.37 were coded as zero (low concentrated racial privilege), values from > − 0.37 to < 0.47 were coded as one (medium concentrated racial privilege), and values from 0.47 to 1 were coded as two (high concentrated racial privilege).

### Health insurance

Percentage of residents without health insurance (range 2.7 to 13.8%) was calibrated based on tertiles where values from 2.7 to 6.5% were coded as zero (low uninsured), values > 6.5–9.7% were coded as one (medium uninsured), and values > 9.7% were coded as two (high uninsured).

### Education

Percentage of residents with college education ranged from 4.5 to 85.6%. We calibrated this condition based on tertiles where values ≤20% were coded as zero (low college education), values > 20 and 40.1% were coded as one (medium college education), and values > 40.1% were coded as two (high college education).

### Limited English proficiency

Percentage of households with limited English proficiency ranged from 0.4 to 25.1%. We calibrated limited English proficiency based on tertiles where values ≤2.2% were coded as zero (low limited English proficiency), values between 2.2 and 4.7% were coded as one (medium limited English proficiency), and values > 4.7% were coded as two (high limited English proficiency).

### Public transportation use

Percentages of residents using public transportation by neighborhood ranged from 9.2 to 45.3%. We calibrated public transportation use based on tertiles where values ≤21.4% were coded as zero (low public transportation use), values between 21.4 and 29.5% were coded as one (medium public transportation use), and values > 29.5% were coded as two (high public transportation use).

### Service employment

The percentage of residents working in service employment ranged from 1.9 to 16.9%. We calibrated service employment based on tertiles where values ≤7.9% were coded as zero (low service employment), values from 7.9 to 9.8% were coded as one (medium service employment), and values greater than 9.8% were coded as two (high service employment).

### Overcrowding

Percentages of overcrowding ranged from 0 to 6.1%. We calibrated overcrowding based on tertiles where values ≤1.7% were coded as zero (low overcrowding), values from 1.7 to 2.4% were coded as one (medium overcrowding), and values > 2.4% were coded as 2 (high overcrowding).

### Meta-factor calibrations

We also created meta-factors using dual calibrations of each factor to identify patterns among potential difference-makers among the conditions. For example, for education, we calibrated a factor called high education where neighborhoods in the highest tertile of education were coded as one, and those not in the highest tertile (low to medium) were coded as zero. We also created a factor called low education where neighborhoods in the lowest tertile of education were coded as one and those not in the lowest tertile (medium to high) were coded as zero.

### Factor selection

To reduce our data and focus our analysis, we implemented a configurational approach to factor selection described in detail elsewhere [26–30]. Briefly, we began by using the “minimally sufficient conditions” routine (i.e., msc function within the R package “cna”) to look across all 43 neighborhoods and all 8 factors at once, comprehensively scanning the entire dataset to identify specific configurations of conditions with strong connections to the outcome of interest (i.e., persistently low COVID-19 vaccination rates). This process exhaustively considered all one-, two- and three-condition configurations instantiated in the dataset, assessed each configuration against a prespecified consistency threshold, retained all configurations that satisfied this criterion, and then generated a “condition table” to list and organize the Boolean output. In a condition table, rows contain all configurations of conditions that meet a specified consistency level while column variables include outcome, conditions, consistency and coverage. We generated the msc routine condition tables by specifying a consistency threshold of 100%; if no configurations met this threshold, we iteratively lowered the specified consistency level by 5 points (e.g., from 100 to 95%, etc.) and repeated the process to generate a new condition table. We continued lowering the consistency threshold until there were at least two potential configurations of neighborhood-level conditions that met the specified consistency level. Using this approach, we inductively analyzed the entire dataset and used the condition table output to identify a subset of candidate factors to use in model development in the next steps of the configurational analysis.

### Model development

We next developed models by iteratively using model-building functions within the R “cna” software package. We assessed candidate models based on their overall consistency and coverage, as well as potential model ambiguity (when competing models satisfy the specified consistency and coverage thresholds and explain the outcome of interest equally well, as reflected by similar consistency and coverage scores). We selected a final model based on the same criteria of overall consistency and coverage, with no model ambiguity. The Coincidence Analysis package (“cna”) in R [29], R (version 3.5.0), and Microsoft Excel were used to support the analyses. Maps were created with ArcGISPro 2.9.1 (ESRI, Redlands CA) using 2010 ZIP Code Tabulation Area (ZCTA) boundaries from the US Census.

## Results

Two factors consistently distinguished neighborhoods with persistently low COVID-19 vaccination rates from the others: education and concentrated racial privilege. The combination of low college education together with low/medium concentrated racial privilege was sufficient to identify neighborhoods with persistently low COVID-19 vaccination rates.

**Fig. 1:**
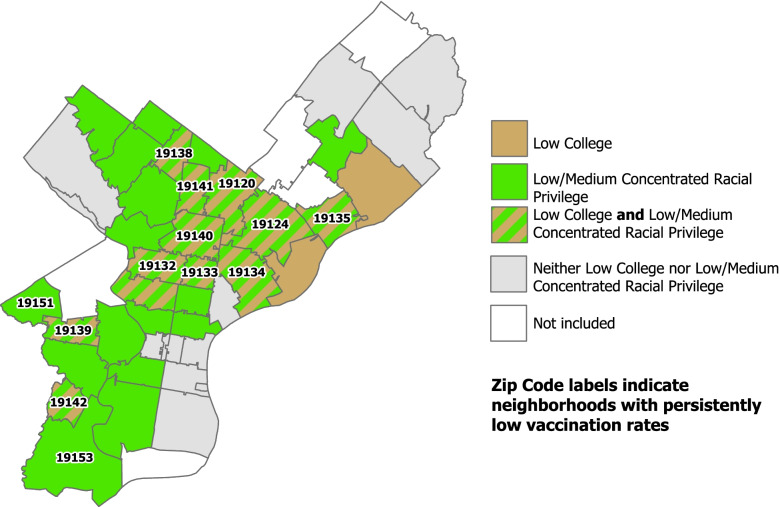
Pathways sufficient for neighborhoods having persistently low COVID-19 vaccination rates by neighborhood

In the geospatial mapping of these findings (see Fig. 1), one can directly observe that all but two of the neighborhoods with persistently low COVID-19 vaccination rates (i.e., the neighborhoods with zip codes listed) have a combination of green and brown, indicating the joint presence of low college education and low/medium concentrated racial privilege. This solution thus explains or “covers” 11 of the 13 neighborhoods with persistently low COVID-19 vaccination rates, translating into a coverage score of .85 (11/13). Furthermore, the solution is highly consistent in distinguishing neighborhoods with persistently low COVID-19 vaccination rates from the others, as there is only neighborhood in the analysis (zip code 19121, directly below 19,132, labeled on the map) that had both conditions present and did not have persistently low COVID-19 vaccination rates, translating into a consistency score of .92 (11/12). Featuring both high coverage and consistency scores, the combination of these two specific conditions proves a crucial difference-maker.

We also explored how levels of college education and/or concentrated racial privilege linked to those 29 neighborhoods where COVID-19 vaccination rates were not persistently low. Medium/high college education by itself was a sufficient condition for not being a neighborhood with persistently low COVID-19 vaccination rates, as was high concentrated racial privilege.

**Fig. 2:**
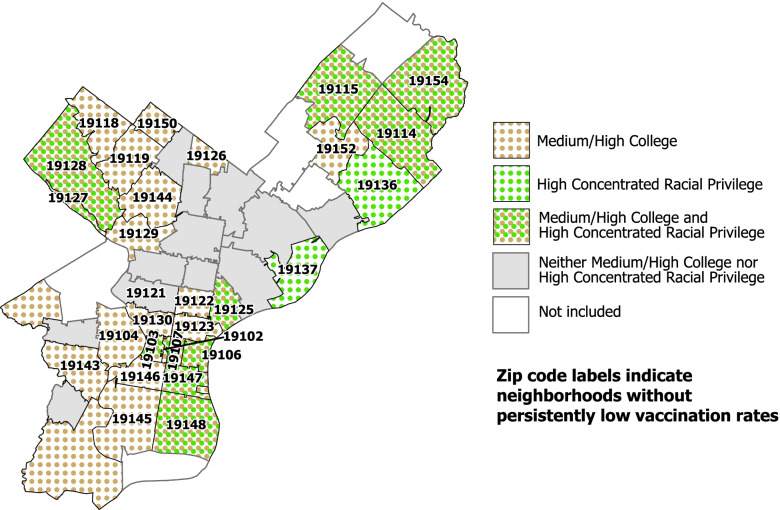
Pathways sufficient for neighborhoods not having persistently low COVID-19 vaccination rates by neighborhood

The geospatial mapping of these findings (see Fig. 2) shows that every neighborhood with medium/high COVID-19 vaccination rates (i.e., the neighborhoods with zip codes listed) has either green dots, brown dots, or a combination of green and brown dots, with only one exception. This solution thus explains or “covers” 28 of the 29 neighborhoods without persistently low vaccination rates, yielding an extremely high coverage score of .97 (28/29). The solution is also highly consistent in distinguishing neighborhoods with medium/high vaccination rates from the others, as there are only two neighborhoods in the analysis that had either condition present and did not have medium/high vaccination rates for a consistency score of .93 (28/30). With both high coverage and consistency scores, the presence of either medium/high college or high concentrated racial privilege are difference-making conditions that uniquely and consistently identified neighborhoods with medium/high COVID-19 vaccination rates.

A table displaying the demographic characteristics of each of these 13 neighborhoods is included in the supplementary materials (See Supplementary Table 1, Additional File 1.) Figures displaying visualizations of neighborhoods by ZIP code with persistently low COVID19 vaccination rates and neighborhoods without persistently low COVID-19 vaccination rates are included in the supplementary materials (See Supplementary Figs. 1 and 2, Additional Files 2 and 3.)

## Discussion

Our study used CCMs, specifically CNA [30], along with geospatial mapping, to identify how multiple neighborhood conditions link to persistently low COVID-19 vaccination rates. A key finding from this study is that while neither low college education nor low/medium concentrated racial privilege alone was linked with persistently low COVID-19 vaccination rates, the presence of both these factors together was consistently linked to this outcome. Our complementary analysis of neighborhoods that do not have persistently low COVID-19 vaccination rates affirms the importance of these same two factors: education and racial privilege. We found that different values of these two factors, in other words medium/high college education and high concentrated racial privilege, were consistently linked to neighborhoods without persistently low COVID-19 vaccination rates, with the presence of either condition alone proving sufficient.

Our study adds a new perspective to how social conditions impact COVID-19 vaccination rates. We found that in eleven of thirteen neighborhoods, the combination of low education and low/medium concentrated racial privilege was consistently linked to persistently low COVID-19 vaccination rates. The finding that the combination of multiple factors was linked to low COVID-19 vaccination rates demonstrates the concept of Boolean conjunction, where multiple conditions together are sufficient to yield an outcome. We found a different pattern for the neighborhoods without persistently low COVID-19 vaccination rates, where either factor alone - medium/high college education or high concentrated racial privilege – was linked to higher COVID-19 vaccination rates. This finding that multiple different factors taken alone were each sufficient to produce the outcome demonstrates the concept of Boolean disjunction.

A key feature of CCMs, including CNA, is causal asymmetry [31]. Causal asymmetry means that the pathway leading to the presence of an outcome (in this case persistently low COVID-19 vaccination rates) can differ from the pathway leading to the absence of the outcome (in this case medium/high COVID-19 vaccination rates). Our analysis found that both factors, level of education and concentrated racial privilege, occurring together, were sufficient to produce persistently low vaccination rates. For the opposite outcome, medium/high COVID-19 vaccination rates, level of education and concentrated racial privilege were each independently sufficient for the outcome. This finding illustrates the idea of causal asymmetry and is the reason that CCMs require separate analyses for the occurrence of an outcome and for its non-occurrence.

Our findings are consistent with previous studies showing that COVID-19 vaccination rates vary by socioeconomic factors within racial/ethnic groups [32, 33]. Studies have affirmed that individuals who identify as Black, and those with lower levels of education are less likely to indicate willingness to receive a COVID-19 vaccination [34]. National polls of those who were high priority for COVID-19 vaccination early in the roll-out found that among other factors, respondents who identified as Black and those with lower education were most likely to report that they did not intend to receive a COVID-19 vaccination. A scoping review of vaccine hesitancy among health care workers and the general public found that lower education was associated with vaccination hesitancy, potentially due to lower past compliance with vaccinations, lower perceived risk of COVID-19 illness or less familiarity with reliable sources of information about COVID-19 vaccines [35]. For example, a national study of vaccine hesitancy showed that African Americans and those with lower levels of education were significantly more likely than others to report that they would not receive the COVID-19 vaccination [36]. This finding has been confirmed by other regional studies [37].

Understanding low vaccination uptake by African Americans must consider the impact of significant mistrust of health care and public health based on past experiences with these systems. Historical medical and public health atrocities, such as the Tuskegee Syphilis Study and the surreptitious appropriation of cells from the body of Henrietta Lacks, have led to deep mistrust for public health messages and medical interventions in Black and brown communities. Beyond these historical abuses, the everyday racism experienced by Black and brown people, such as having their pain disregarded by health care providers or lack of providers who share their background, may play as great a role in reluctance to seek out vaccinations or other preventive interventions [38]. At the neighborhood level, such everyday racism included lack of neighborhood pharmacies where COVID-19 vaccines were available and lack of outreach to Black and brown communities by health systems. In Philadelphia, it was this very lack of health care access that spurred the launch of the Black Doctors COVID Consortium to address issues of access and mistrust by providing COVID testing and vaccinations, administered by health care providers of color at community sites, including churches [39].

Since COVID-19 vaccination were first offered in late 2020 in the US, studies are still emerging to examine how factors like residential segregation and education impact vaccination uptake, including boosters. Despite this, numerous studies have shed light on the ways that racial segregation can affect health with respect to other health conditions, and these same mechanisms and long-standing structural biases are likely playing a role in COVID-19 vaccinations. For example, residential segregation is known to have a strong link to poverty, poor health, and lack of access to health care [40–42]. Williams and Collins have found that segregation exerts its negative effect on individual and neighborhood health not only through socioeconomic status, but also through the effects of place, shaping health behaviors, poorer access to medical care, neighborhood quality and crime, and lower quality education [42]. Beyond its direct impact on access to health care, residential racial segregation degrades the quality of schools, causing both individual and community-wide impacts [42].

Low levels of education are also known to have a strong association with non-COVID-19 related poor health outcomes, which may suggest structural causes. Kawachi and others have suggested that at the individual level, education provides general knowledge that may be helpful in preventing disease, but that it also confers greater status and greater access to jobs that pay well and have fewer safety hazards [43]. The findings in our study align with other research demonstrating that low levels of education negatively impact vaccine acceptance and health status overall at the individual level [44–46] and at the community level [47].

A strength of our study is the ability to uncover complexity using CNA, a novel configurational analytic method. This method reveals findings that are complementary to results from studies using correlational methods and helps to uncover qualitative differences between neighborhoods that may be important to designing interventions. CCMs are particularly designed for small-N, case-based studies. In this regard, they can be a powerful way to bridge qualitative and quantitative data, and to identify patterns where “bundles” of conditions are necessary to produce an effect. CCMs can also account for the fact that the presence of specific factors may be sufficient for an outcome, but that a pathway may also require the absence of a specific factor. CCMs also allow for the possibility of multiple pathways to an outcome (called equifinality) and causal asymmetry, the property that the pathways to the occurrence of an outcome, in this case persistently low COVID-19 vaccination rates, may be different from the pathways to the absence of the outcome, in this case medium/high COVID-19 vaccination rates. To our knowledge, this is the first study to apply CCMs, specifically CNA, to a geospatial analysis of the important public health and health inequity challenge of low COVID-19 vaccination rates. It is our hope that future studies seeking to understand health disparities will employ similar methods.

Potential alternatives to CCMs that would also allow for the complexity of interaction factors include clustering approaches (parametric, such as finite mixture modeling, or non-parametric such as k-means clustering). However, cluster analysis and CCMs have two differing objectives, as cluster analysis aims to discover groups of observations that are similar to each other (e.g., neighborhoods), while CCMs aims to understand combinations of factors that are associated with an outcome [48]. Quintana’s 2022 review of complexity in social sciences identified three approaches – interaction analysis, structural analysis and CCMs – that are designed to examine the dependencies among causal factors. He noted that interaction analysis focuses on whether the direction and magnitude of a causal effect differs across subgroups, and structural analysis seeks to uncover the causal connections among the variables in particular system. CCMs, by contrast, examine whether specific combinations of factors are sufficient or necessary conditions for generating an outcome [49].

We selected CCMs because they can be applied to case studies with relatively small numbers of cases. In addition, CCMs possess several key features relevant to this analysis. CCMs allow for multiple pathways to an outcome, a feature known as equifinality. Another feature of CCM analysis is asymmetry, meaning that the pathway leading to the presence of an outcome can differ from the pathway leading to the absence of the outcome. These features allowed us to understand how various social factors combined to produce neighborhood outcomes of low COVID-19 vaccination rates. We believe that these findings have implications for how we address this important health inequity.

Our findings align with the concept of intersectionality, a framework for understanding “how multiple social identities such as race, gender, sexual orientation, SES, and disability intersect at the micro level of individual experience to reflect interlocking systems of privilege and oppression (i.e., racism, sexism, heterosexism, classism) at the macro social structural level [44].” Original concepts of intersectionality emerged from feminist theory and increasingly, its theoretical and methodological concepts are being applied to the ways that public health scholars study complex health inequities. As the conceptual and methodological applications of intersectionality continue to grow, a number of scholars have called for intersectional approaches to address pressing health equity challenges such as COVID-19 [50, 51]. In our study, by emphasizing the ways that disadvantages and advantages intersect, we are able to paint a more complex picture in ways that improve our ability to diagnose and address social inequities [16, 52].

Our study has several weaknesses. One weakness of this study is the use of vaccination data at the zip code tabulation area level. ZCTAs (and ZIP Codes) represent large and relatively heterogeneous geospatial units of analysis which do not necessarily correspond to the neighborhoods that residents might define, nor do they drill down to the level of census tracts. In addition, our sample includes a total of 43 neighborhoods. While CNA is routinely applied to small-N case studies with 10–100 cases, the higher the sample size, the better the performance of the method [53]. Consistency and coverage are the core parameters of fit for CCMs and in our study, both parameters were excellent. Future research performed at the census tract level or at the community resident-defined neighborhood level will help us to better understand complex drivers of health inequities, while also increasing the sample size for the analysis. Another potential limitation is that while our data refers to neighborhood (ZCTA) of residence, people vaccinated in other states may not be included in this dataset due to the lack of federal coordination in vaccination data systems.

In Philadelphia from December 16, 2020, through April 12, 2021, eligibility for COVID-19 vaccinations was limited to higher risk groups, such as health care workers, essential workers, those over the age of 65, and those with chronic conditions. On April 16, 2021, eligibility was expanded to include all residents 16 years of age or older. This raises the potential limitation that neighborhoods with persistently low COVID-19 vaccination rates might have had a lower proportion of eligible individuals than other neighborhoods. While we did not include factors reflecting proportions of populations in various high-risk groups, we found that that the neighborhood factors that were linked with persistently low COVID-19 vaccination rates in March and April were also linked to persistently low COVID-19 vaccination rates in May after eligibility was expanded to all residents.

We included factors that related to COVID-19 infection rates and mortality at the neighborhood level in previous studies. It is possible that other factors relating to COVID-19 vaccination rates in specific (e.g., trust in governmental institutions or ideology) [41] and which might be revealed through in-depth qualitative studies, were not included in this analysis. Nonetheless, the combinations of conditions and the pathways identified here were able to identify neighborhoods with low COVID-19 vaccination rates with high consistency and coverage. Future studies using qualitative and quantitative methods will be useful in defining the underlying mechanisms necessary for intervention.

## Conclusions

A practical policy implication of these findings is that neighborhoods that have low rates of COVID-19 vaccination and other pressing health disparities, may require strategies designed to address multiple intersecting factors. For example, in neighborhoods where low levels of education combine with low concentrated racial privilege, health enhancing interventions should focus on improving access to higher education while also dismantling the structural racism underlying residential segregation. Approaches that use CCMs to identify geospatial patterns, which might best be termed “geoconfigurational,” represent a unique approach to understanding health inequities at the neighborhood level. This approach affirms the credo that “social policy is health policy.” The complex interplay of specific social vulnerabilities must be better understood and addressed with the same vigor as individually focused interventions aimed at increasing COVID-19 vaccination rates. Future research should also shed light on how these pathways relate to disparities in other health outcomes, such as violence and chronic disease. The results of such studies could guide public health policymakers and community leaders in addressing the challenges that are most specific to their communities.

## Supplementary Information

**Additional file 1.**

**Additional file 2.**

**Additional file 3.**

## Data Availability

Datasets and code for replication are available at https://github.com/JohnARich/CNA_COVIDVax_Philly

## Abbreviations

CCM: Configurational comparative method
CNA: Coincidence analysis
ICE-Income: Index of Concentration at the Extremes - Income
ICE-BlackNH: Index of Concentration at the Extremes - Non-Hispanic Black
COVID-19: Coronavirus disease 2019
