# Supplementary material for: How education and racial segregation intersect in neighborhoods with persistently low COVID-19 vaccination rates in Philadelphia
Source: BMC Public Health. 2022 May 25;22:1044. doi: 10.1186/s12889-022-13414-3 (PMC9130689; doi:10.1186/s12889-022-13414-3)
Supplement: Supplementary file 1 — Additional file 1. [file 12889_2022_13414_MOESM1_ESM.pdf]

Supplementary Table 1, Additional File 1

Demographic Characteristics of Neighborhoods with Persistently Low COVID-19 Vaccination Rates

Neighborhood ZIP Code	Percent without Health Insurance	Percent with any College	Percent using Public Transportation	Percent working in the Service Industry	Percent of Households with >1 person/room	Percentage of Households with Limited English Proficiency	Index of Concentration at the Extremes for Black Non-Hispanic Race	Index of Concentration at the Extremes for Income	March18 COVID-19 Vaccination Rate/10,000 Residents	April18 COVID-19 Vaccination Rate/10,000 Residents	May18 COVID-19 Vaccination Rate/10,000 Residents
19120	13.80%	13.80%	22.30%	9.10%	3.60%	14.90%	-0.44	-0.23	1018	2034	2964
19124	12.20%	10.70%	24.80%	10.20%	3.00%	11.10%	-0.23	-0.29	979	1932	2859
19132	8.60%	9.60%	43.10%	9.30%	1.70%	1.20%	-0.87	-0.52	1319	2370	3281
19133	13.20%	4.50%	28.70%	11.20%	5.90%	25.10%	-0.31	-0.55	984	1979	2803
19134	9.70%	11.00%	21.50%	8.70%	6.10%	13.70%	0.19	-0.33	1090	2023	2922
19135	10.30%	13.40%	20.00%	12.50%	2.40%	4.20%	0.21	-0.15	1180	2025	2910
19138	8.60%	15.40%	32.10%	12.00%	1.00%	1.20%	-0.89	-0.23	1394	2350	3147
19139	10.80%	15.30%	45.30%	9.40%	2.30%	1.40%	-0.81	-0.41	1351	2216	3016
19140	11.30%	7.30%	39.10%	8.80%	3.00%	11.90%	-0.49	-0.50	1098	2257	3193
19141	10.20%	18.00%	37.20%	11.70%	2.50%	3.90%	-0.80	-0.31	1122	1989	2728
19142	11.40%	9.60%	39.20%	10.90%	3.60%	7.50%	-0.78	-0.37	949	1815	2754
19151	8.70%	24.50%	34.80%	6.60%	2.20%	2.60%	-0.81	-0.05	1366	2161	2831
19153	7.70%	22.00%	28.20%	10.40%	1.70%	4.00%	-0.61	-0.14	1458	2492	3338
