# Supplementary material for: How education and racial segregation intersect in neighborhoods with persistently low COVID-19 vaccination rates in Philadelphia
Source: BMC Public Health. 2022 May 25;22:1044. doi: 10.1186/s12889-022-13414-3 (PMC9130689; doi:10.1186/s12889-022-13414-3)
Supplement: Supplementary file 3 — Additional file 3. [file 12889_2022_13414_MOESM3_ESM.pdf]

Supplementary Figure 2, Additional File 2.

Solution visualization for neighborhoods by zip code that did not have persistently low COVID19 vaccination rates.

OUTCOME_PERSISTENTLYLOW	LOW_COLLEGE	HIGH_CONCENTRATED_RACIAL_PRIVILEGE	ZIPCODE
0	0	0	19122
0	0	0	19152
0	0	0	19104
0	0	0	19145
0	0	0	19130
0	0	0	19123
0	0	0	19118
0	0	0	19129
0	0	0	19119
0	0	0	19146
0	0	0	19144
0	0	0	19126
0	0	0	19143
0	0	0	19150
0	0	1	19154
0	0	1	19114
0	0	1	19148
0	0	1	19115
0	0	1	19127
0	0	1	19147
0	0	1	19107
0	0	1	19125
0	0	1	19128
0	0	1	19102
0	0	1	19103
0	0	1	19106
0	1	1	19136
0	1	1	19137
0	1	0	19121
1	1	0	19124
1	1	0	19133
1	1	0	19134
1	1	0	19135
1	1	0	19142
1	1	0	19120
1	1	0	19140
1	1	0	19141
1	1	0	19139
1	1	0	19132
1	1	0	19138
1	0	0	19151
1	0	0	19153

Dotted red line separates cases with and without outcome; yellow highlighter indicates consistent cases; green highlighter indicates inconsistent cases.
